# Urinary Metal Levels after Repeated Edetate Disodium Infusions: Preliminary Findings

**DOI:** 10.3390/ijerph17134684

**Published:** 2020-06-29

**Authors:** Zenith H. Alam, Francisco Ujueta, Ivan A. Arenas, Anne E. Nigra, Ana Navas-Acien, Gervasio A. Lamas

**Affiliations:** 1 Department of Medicine, Columbia University, Mount Sinai Medical Center, 4300 Alton Road, Miami Beach, FL 33140, USA; zenith.haq@gmail.com (Z.H.A.); francisco.ujueta@msmc.com (F.U.); 2 Division of Cardiology, Columbia University, Mount Sinai Medical Center, 4300 Alton Road, Miami Beach, FL 33140, USA; ivan.arenas@msmc.com; 3 Department of Environmental Health Sciences, Columbia University Mailman School of Public Health, 722 West 168th Street, New York, NY 10032, USA; aen2136@cumc.columbia.edu (A.E.N.); an2737@cumc.columbia.edu (A.N.-A.)

**Keywords:** lead, cadmium, chelation, vascular disease, atherosclerosis

## Abstract

Environmentally acquired lead and cadmium are associated with increased cardiovascular disease risk. In the Trial to Assess Chelation Therapy, up to 40 infusions with edetate disodium over an approximately one-year period lowered the cardiovascular disease risk in patients with a prior myocardial infarction. We assessed whether a reduction in surrogate measures of total body lead and cadmium, post-edetate disodium urine lead and pre-edetate urine cadmium, could be detected after repeated edetate disodium-based infusions compared to the baseline. Fourteen patients with coronary artery disease received multiple open-label edetate disodium infusions. The urine metals pre- and post-edetate infusion, normalized for urine creatinine, were compared to urine levels pre and post final infusion by a paired *t*-test. Compared with the pre-edetate values, post-edetate urine lead and cadmium increased by 3581% and 802%, respectively, after the first infusion. Compared to baseline, post-edetate lead decreased by 36% (*p* = 0.0004). A reduction in post-edetate urine lead was observed in 84% of the patients after the final infusion. Pre-edetate lead decreased by 60% (*p* = 0.003). Pre-edetate lead excretion became undetectable in nearly 40% of patients. This study suggests that edetate disodium-based infusions may decrease the total body burden of lead. However, our data suggest no significant reduction in the body burden of cadmium.

## 1. Introduction

Environmentally acquired metals have been associated with increased cardiovascular disease risk in epidemiologic studies [1,2,3]. The CDC’s Agency for Toxic Substances and Disease Registry (ATSDR) Substance Priority List has listed lead as the second greatest threat to health via toxicity and exposure potential since 1997 [4]. Although environmental contamination has decreased with the elimination of lead in gasoline, chronic contamination exists via water pipes, lead-based paints, auto batteries, contaminated soil, and airborne emissions [4,5]. Lead accumulates within the mineralizing tissues (bones, teeth) with a half-life of 20–30 years. As a result, body stores of lead contribute to chronic exposure, as they leach out of the bone, even when new exposure is controlled [6]. The ATSDR lists cadmium in seventh place as a toxic substance of concern [4]. This toxic metal, which accumulates in fertilizer and green leafy plants, enters the body through tobacco use, ingested grains and vegetables, and the consumption of organ meats. Additionally, indirect uptake occurs through its industrial use (rechargeable batteries, plastic stabilizer, and construction) [7]. Cadmium accumulates in soft tissues (e.g., kidneys, liver) and its half-life in the body ranges between 6 and 38 years [4]. A recent systematic review concluded that chronic cadmium exposure is an independent risk factor for vascular disease, especially peripheral artery disease (PAD) and coronary artery disease (CAD) [7,8]. Moreover, preliminary evidence suggests that the severity of PAD may be associated with higher cadmium levels in both spontaneous and stimulated (post-edetate disodium) urine, showing the highest cadmium levels in patients with critical limb ischemia (CLI) [8]. Uncontrolled observational studies of edetate disodium infusions leading to wound healing and improvement in quality of life in patients with CLI have been published [9,10].

The total body burden of lead, measured non-invasively by using K-shell X-ray fluorescence, correlates with clinical outcomes, but this equipment is not widely available [11]. The total body burden of lead may also be estimated by post-chelation urine lead, while the total body burden of cadmium can be estimated by pre-chelation, or spontaneous, urine cadmium [12,13]. Edetate disodium is a polydentate chelator with particular affinity for cations with a +2 charge, including lead and cadmium [3]. Arenas et al. demonstrated that a single infusion of 3 g of edetate disodium leads to an approximately 4000% increase in excreted lead, 600% increase in excreted cadmium, and 400% increase in excreted nickel within 24 h [14]. However, the effect of repetitive treatments of edetate disodium chelation on lead and cadmium body burden is unknown. This analysis evaluated the effect of repeated edetate disodium chelation infusions (range 22 to 61 edetate disodium infusions) on surrogate markers for lead and cadmium body burden (post-chelation urine lead and pre-chelation urine cadmium). Lead and cadmium are associated with increased cardiovascular disease risk, and we postulate that decreasing the total body burden of each metal may lead to decreased cardiovascular mortality [2,3,7]. Additionally, as prior studies revealed that arsenic, nickel, and mercury are associated with atherosclerosis, the effect of edetate disodium on the pre- and post-chelation levels of these metals/metalloids is also reported [2,15,16].

## 2. Materials and Methods

### 2.1. Methodology

This is an exploratory retrospective study of 14 patients with CAD without occupational exposure to toxic metals. Patients younger than 50 years old, myocardial infarction (MI) <6 weeks prior to the study, or creatinine levels of >2.0 mg/dL were excluded [3,17,18,19,20]. Eligible patients participated in prior studies of edetate disodium infusions for vascular disease or received it based on clinical indications [3,17,18,19,20,21].

### 2.2. Materials

The Mount Sinai Institutional Review Board approved the retrospective chart review for the analyses of all patients receiving edetate disodium-based therapy for the treatment of atherosclerosis. The infusion regimen and dosing schedule mirrored the one approved for study under an Investigational New Drug application to the US Food and Drug Administration [3,17,18,19,20]. It consisted of up to 3 g of edetate disodium adjusted downward for decreasing renal function, 7 g of ascorbic acid, 2 g of magnesium chloride, 100 mg of procaine hydrochloride, 2500 units of unfractionated heparin, 2 mEq potassium chloride, 840 mg sodium bicarbonate, 250 mg of pantothenic acid, 100 mg of thiamine, and 100 mg of pyridoxine infused slowly over three or more hours [3,17,18,19,20]. Additionally, patients received a daily regimen of vitamins and minerals to supplement the potential depletion by edetate disodium including vitamin B6 25 mg, zinc 25 mg, copper 2 mg, manganese 15 mg, and chromium 50 µg [3,17,18,19,20]. Patients were selected to whom edetate disodium had been administered intravenously on a weekly basis for at least 2 weeks. Patients who received only one infusion were excluded. At the first and final infusion, urine was collected pre and post infusion in metal-free containers, assayed with inductively coupled plasma-mass spectrometry (ICP-MS; Doctor’s Data, St. Charles, IL, USA), and controlled for urine concentration by expressing results as micrograms of metal per gram of creatinine [3,17,18,19,20]. The lower limits of detection were 0.07 µg/g for lead, 0.07 µg/g for cadmium, 0.28 µg/g for mercury, and 0.56 µg/g for nickel. Although this report focuses on two highly atherogenic metals, lead and cadmium, we also report data for other toxic metals and metalloids associated with atherosclerosis (arsenic, mercury, and nickel) that were measured as part of the multielement laboratory panel (Table 1).

### 2.3. Statistical Analyses

Metal levels are presented as means and standard deviations (SD). The pre and post metal levels were parametric in the log scale. Therefore, a paired *t*-test with SPSS version 20 (Armonk, NY, USA) was used to compare metal levels in pre- and post-chelated urine at baseline (pre-treatment) and after chronic treatment with edetate disodium infusions. In addition, for more confirmation that the results were robust, a non-parametric Kruskal–Wallis test was performed, with similar results. Power analysis was not performed. Undetectable metal values (0.1% for lead, 0.1% for cadmium, none for arsenic, 0.4% mercury, and 0.8% nickel) were replaced by the lower limit of detection divided by the square root of two. Figures were constructed in R version 3.5.3. (Auckland, New Zealand).

## 3. Results

The mean (SD) age of the patients was 74 (11, range 55 to 90) years and mean (SD) serum creatinine was 0.9 (0.3) mg/dL. Ten (71%) patients were male and 7 (50%) were Hispanic. The mean baseline Modification of Diet in Renal Disease (MDRD) eGFR was 78 mL/min/1.73 m [22]. All patients had CAD; 12 (86%) had diabetes, 12 (86%) had PAD, and 6 (43%) had a smoking history (Table 2). Patients received a median of 51 infusions (range 22 to 61).

In an exploratory manner, we conducted stratified analyses by smoking status and race/ethnicity. At the final treatment visit, pre- versus post-edetate change in urine lead was not meaningfully different for Hispanic patients (4435%, *n* = 7) or non-smoking patients (4446%, *n* = 8) compared to all patients (4730%).

**Figure 1:**
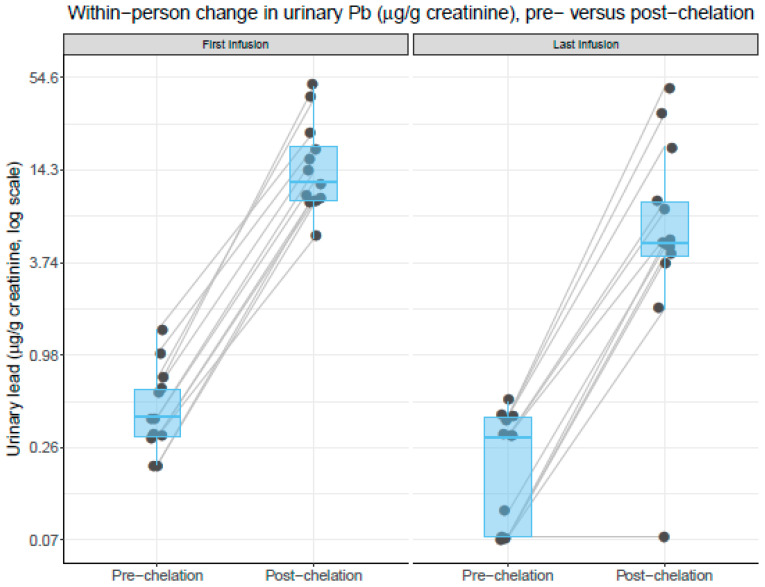
Within-person change comparing pre versus post edetate chelation urinary lead concentrations (µg/g of creatinine). All values are controlled for creatinine concentration. Results are presented separately for the first (baseline) and last (final) infusion visits. Each black dot (jittered) represents an individual patient measurement. Gray lines connect measurements from individual patients and indicate a within-person change in urinary lead concentrations. Boxplot upper, middle, and lower hinges correspond to the 25th, 50th, and 75th percentiles, respectively. The left panel compares the within-person change in the urinary lead concentrations in urine samples collected at the first (baseline) visit, comparing pre versus post edetate chelation. The left panel compares the within-person change in urinary lead concentrations in urine samples collected at the last (final) visit, comparing pre versus post edetate chelation. Urinary lead concentrations are plotted on a log scale. The blue horizontal bars represent the median values.

**Figure 2:**
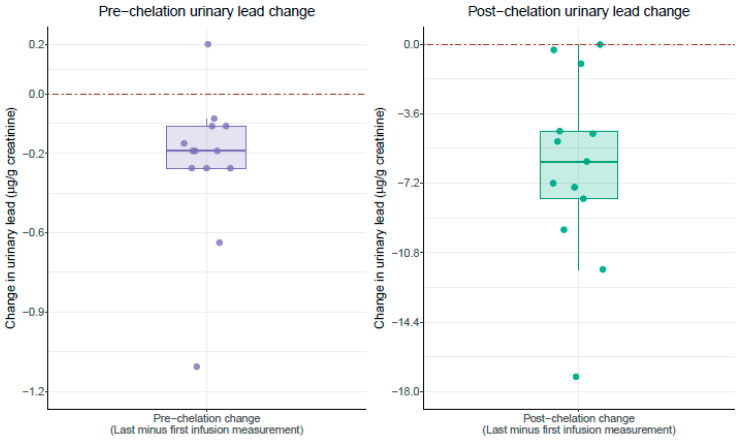
Effect of chelation therapy on patient urinary lead concentrations (µg/g of creatinine). All values are controlled for creatinine concentration. Each dot represents the within-person change in urinary lead concentrations (µg/g of creatinine) comparing urine collected at the last (final) infusion minus urine collected at the first (baseline) infusion for an individual patient. The left panel compares the within-person change in the urinary lead concentrations (µg/g of creatinine) for urine samples collected pre edetate chelation. The right panel compares the within-person change in urinary lead concentrations (µg/g of creatinine) for urine samples collected post edetate chelation. Boxplot upper, middle, and lower hinges correspond to the 25th, 50th, and 75th percentiles, respectively. The red dashed line indicates zero (no change).

**Figure 3:**
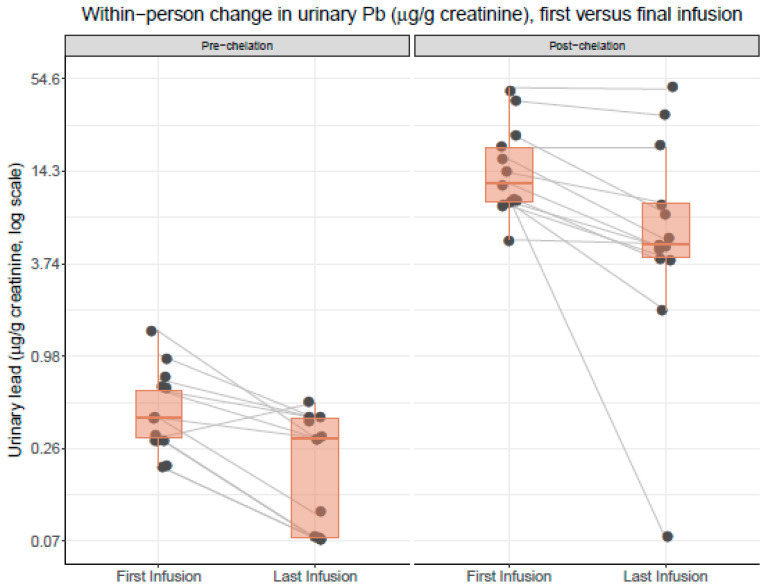
Within-person change in urinary lead concentrations (µg/g of creatinine) comparing the first and last infusion visits. Results are presented separately for urinary lead concentrations measured pre versus post edetate chelation. All values are controlled for creatinine concentration. Each black dot (jittered) represents an individual patient measurement. Gray lines connect measurements from individual patients and indicate a within-person change in the urinary lead concentrations. Boxplot upper, middle, and lower hinges correspond to the 25th, 50th, and 75th percentiles, respectively. The left panel compares the within-person change in the urinary lead concentrations in urine samples collected pre edetate chelation, comparing the first (baseline) and last infusion visits. The right panel compares the within-person change in the urinary lead concentrations in urine samples collected post edetate chelation, comparing the first (baseline) and last infusion visits. Orange horizontal bars represent the median values.

At baseline, all patients had detectable pre-edetate urine levels of lead and cadmium. After the initial infusion, post-edetate urine lead increased by 3581% (range 1667% to 7900%) and cadmium by 802% (range 250% to 1550%) (Table 1, Figure 1). After the final (last) infusion, the average urinary lead concentration increased by 4730% (Figure 1). A reduction in post-edetate urine lead was observed in 84% of the patients after the final treatment (Figure 2). The two patients (16%) who did not have diabetes did not experience a reduction in post-edetate urine lead. Post-edetate urine lead, the surrogate marker of body burden, decreased by 36% (*p* = 0.0004) compared with the baseline. Pre-edetate, spontaneous urine lead decreased by 54% (*p* = 0.006). Moreover, spontaneous, pre-edetate urine lead became undetectable (≤0.07 µg/g of creatinine) in nearly 40% of patients after their infusion regimen (Figure 3). Post-edetate urine lead became undetectable in 7% of patients. No statistically significant differences were found for pre- or post-edetate urine cadmium, nickel, arsenic, or mercury compared with the baseline (Table 1).

## 4. Discussion

Cardiovascular disease prevention extends beyond behavioral modifications and conventional medical therapy. Indeed, even when accounting for all traditional cardiovascular risk factors, up to 30% of atherosclerotic or coronary events cannot be explained. This observation suggests that there may be additional environmentally acquired, and potentially modifiable, coronary risk factors [3,17,18,19,20,21,23]. Lead, cadmium, nickel, mercury, and arsenic are associated with cardiovascular disease and premature mortality [2,4,7,15,16]. Recent studies suggest that pharmacotherapy with metal chelating agents may reduce recurrent cardiovascular events [3,17,18,19,20]. However, it is unknown if this beneficial cardiovascular effect is due to a reduction of toxic metal burden or other mechanisms.

### 4.1. Edetate Disodium and Patient Safety

Edetate disodium therapy, at large doses, may induce renal toxicity, most commonly in patients with underlying renal disease. As patients with creatinine levels of >2.0 mg/dL were excluded from our study, there was no change in renal function and no adverse effects reported [3,17,18,19,20].

Hypocalcemia was not observed in our study, as it occurs principally with the rapid infusion of edetate disodium. Our study utilized the infusion protocol from Trial to Assess Chelation Therapy (TACT). Infusions were conducted over a three-hour period and serial labs were obtained to ensure adequate electrolyte levels. [3,17,18,19,20]. Additional side effects including metal deficiency syndromes, arrhythmias, respiratory arrest, hypoglycemia, or hematological abnormalities were not observed [3,17,18,19,20]. The regimen of edetate disodium therapy appeared to have been well-tolerated in this small patient population.

The tolerability of the infusion regimen is unsurprising. TACT enrolled 1708 patients with a history of MI and administered a total of 55,222 infusions—27,382 infusions of which were edetate disodium based. There were no statistically significant differences in major or minor adverse events of edetate disodium compared with placebo infusions [3,17,18,19,20].

### 4.2. Decrease in Lead Burden

The present exploratory study suggests that a regimen of repeated edetate disodium infusions may decrease a potential surrogate marker of total body burden for lead—a cardiotoxic agent. For cadmium and mercury, no significant decline was observed. We observed the baseline urine lead levels in all patients studied. Post-edetate urine lead decreased in all but 2 (16%) subjects. Interestingly, these 2 subjects were the only ones without diabetes (Table 2). Escolar et al. analyzed a pre-defined subset of patients with diabetes from TACT and reported a reduction in cardiovascular events and all-cause mortality [3,17,18,19,20]. TACT2 will explore whether a reduction in post-infusion urine lead is associated with a reduction in clinical events.

## 5. Conclusions

Toxic metal exposure remains an under-recognized contributor to vascular disease [1,2,9,23]. Animal models have shown that lead may increase oxidative stress by increasing the production of reactive oxygen species, which interferes with the nitric oxide signaling cascade, leading to endothelial dysfunction, and eventually atherosclerotic disease burden [18]. Lead concentrations as low as 5 µg/dL, and possibly lower, are associated with increased cardiovascular disease mortality. Thus, there is no known “safe” lead level [1]. In a meta-analysis, Chowdhury et al. reported that lead and cadmium burden has a dose–response relationship with cardiovascular disease risk [2]. The pooled adjusted relative risks were 1.43 for incident cardiovascular disease and 1.85 for incident coronary artery disease [2]. A single edetate disodium infusion effectively chelated and removed large amounts of two important vasculotoxic metals, lead and cadmium. However, whether repeated chelation can effectively reduce total body metal burden is unknown. Our preliminary study in patients with cardiovascular disease supports that edetate-disodium-based chelation decreased our surrogate marker of lead burden but failed to significantly reduce cadmium, nickel, and mercury measurements. Additional research with larger sample size, control group, and long-term follow up for cardiovascular and all-cause mortality is in progress.

### Limitations

This is a small study that used surrogate markers of lead and cadmium body burden. There was no control group; each patient acted as their own control. Blood metals or specific biomarkers for cumulative lead exposure (e.g., X-ray fluorescence or whole-blood DNA methylation profiles [11,24]) were also not measured. The number of edetate disodium infusions and the relationship between pre and post edetate disodium urine metal levels were not assessed. Therefore, a conclusion regarding a dose-dependent relationship could not be drawn. We were unable to assess potential differences in treatment response by patient characteristics because of the small sample size.

However, these preliminary studies support conducting larger studies of patients, including those with a history of myocardial infarction (TACT2, www.tact2.org) and critical limb ischemia (TACT3a), which are in progress.

## Figures and Tables

**Table 1:** Pre-chelation and post-chelation metals at the first and final infusion: Mean urinary metal levels ± SD of lead (Pb), cadmium (Cd), nickel (Ni), arsenic (As), and mercury (Hg) expressed in µg/g of creatinine in patients pre-chelation (left) and post-chelation (right) for the first and final infusions.

	Pre-Chelation			Post-Chelation		
Metals	First | Final Infusion	Percent Change	*p*-Value	First | Final Infusion	Percent Change	*p*-Value
	(µg/g of Creatinine)			(µg/g of Creatinine)		
Pb	0.5 ± 0.4 | 0.2 ± 0.2	−54.5%	0.006	17. ± 13.0 | 11.2 ± 13.8	−36.3%	0.0004
Cd	0.4 ± 0.2 | 0.4 ± 0.5	−7.7%	0.86	3.1 ± 1.5 | 2.9 ± 1.7	−6.7%	0.65
Ni	3.6 ± 2.3 | 3.8 ± 2.9	4.5%	0.85	7.4 ± 3.2 | 6.2 ± 2.7	−15.9%	0.24
As	23.2 ± 13.7 | 47.1 ± 6.6	102%	0.21	19.8 ± 13.3 | 50.2 ± 64.6	153%	0.12
Hg	0.8 ± 0.69 | 0.85 ± 0.8	11%	0.79	0.40 ± 0.5 | 0.42 ± 0.4	3.8%	0.91

**Table 2:** Baseline characteristics: Patients (*n* = 14) age, sex (male (M) or female (F)), and race. Additionally, a history of coronary artery disease (CAD), peripheral artery disease (PAD), diabetes, and smoking is indicated with a yes (Y) or no (N).

Baseline Characteristics						
Age	Sex	Race	CAD	PAD	Diabetes	Smoking
86	M	Hispanic	Y	Y	N	N
69	M	Hispanic	Y	Y	Y	N
68	M	Caucasian	Y	Y	Y	Y
55	M	Caucasian	Y	Y	Y	Y
56	M	Caucasian	Y	N	Y	Y
76	F	Caucasian	Y	Y	N	Y
84	M	Caucasian	Y	Y	Y	N
90	F	Hispanic	Y	Y	Y	Y
80	M	Caucasian	Y	Y	Y	N
83	F	Hispanic	Y	Y	Y	Y
69	F	Hispanic	Y	Y	Y	N
82	M	Hispanic	Y	Y	Y	N
64	M	Caucasian	Y	N	Y	N
75	M	Hispanic	Y	Y	Y	N

## Data Availability

The data that support the findings of this study are available from the corresponding author upon reasonable request.

## Abbreviations

TACT	Trial to Assess Chelation Therapy
CDC	Center for Disease Control
CAD	coronary artery disease
CLI	critical limb ischemia
PAD	peripheral artery disease
ATSDR	Agency for Toxic Substances and Disease Registry
MI	myocardial infarction
